# Mistakes that matter: An event-related potential study on obsessive-compulsive symptoms and social performance monitoring in different responsibility contexts

**DOI:** 10.3758/s13415-020-00796-3

**Published:** 2020-05-06

**Authors:** M. Jansen, E. R. A. de Bruijn

**Affiliations:** 1 Department of Clinical Psychology, Institute of Psychology, Leiden University, Wassenaarseweg 52, 2333 AK Leiden, The Netherlands; 2 Leiden Institute for Brain and Cognition (LIBC), Leiden, The Netherlands

**Keywords:** Error-related negativity, Event-related potential, Social performance monitoring, Obsessive-compulsive symptoms, Responsibility

## Abstract

Mistakes that affect others often are linked to increased feelings of responsibility and guilt. This especially holds for individuals high in obsessive-compulsive symptoms (OCS), who are characterized by inflated feelings of responsibility and a fear of causing harm to others. This event-related potential study investigated individual differences in OCS in social performance monitoring with a focus on the role of responsibility for other’s harm and the error-related negativity (ERN). Healthy volunteers low (N = 27) or high (N = 24) in OCS performed a Flanker task in the presence of a gender-matched peer in three conditions. Mistakes could either have negative monetary consequences for 1) oneself, 2) the other, or 3) no one. Results showed enhanced ERNs for mistakes that harmed others instead of the self for individuals high in OCS, whereas individuals low in OCS showed decreased amplitudes specifically for mistakes affecting no one versus oneself. Amplitudes of the error positivity but not the ERN also were larger in the high OCS group. These findings indicate that high OCS are associated with enhanced performance monitoring in a social responsibility context, when mistakes harm others instead of the self, and demonstrate the importance of integrating the social context in performance monitoring research as a way to shed more light on obsessive-compulsive symptomatology.

**Electronic supplementary material:**

The online version of this article (10.3758/s13415-020-00796-3) contains supplementary material, which is available to authorized users.

To detect our mistakes and learn from them, we need to monitor our performance continuously. As such, performance monitoring helps us to behave in a safe, flexible, and adaptive way. However, our behavior often takes place in a social context, and hence our mistakes may not only affect ourselves but also the people around us. Mistakes made in a social context therefore often are linked to increased feelings of responsibility and guilt. This is especially the case for individuals who score high on obsessive-compulsive symptoms (OCS). Obsessive-compulsive disorder (OCD) is a prevalent and highly debilitating disorder characterized by obsessions, i.e., intrusive and unwanted thoughts, and compulsions, which are repetitive ritualistic behaviors or mental acts that individuals feel driven to perform (American Psychiatric Association, 2013). The disorder has a considerable social component, because individuals with OCD often are characterized by an inflated sense of responsibility together with a fear of making mistakes that may harm others (Hezel & McNally, 2016). For example, people with OCD may repeatedly check light switches, electronic devices, (gas) taps, and locks to make sure that family members are protected from accidents, such as fire or burglaries. In other instances, they may repeatedly check their car for damage to make sure that they did not accidentally hit someone while driving. Other patients may engage in rituals, such as counting to a certain number to neutralize the fear that something bad will happen to a loved one if a ritual is not performed. This inflated sense of responsibility and fear of causing harm also is observed in nonclinical samples scoring high on OCS (Gibbs, 1996). Yet, previous studies investigating obsessive-compulsive symptomatology have been limited to performance monitoring processes in an individual, i.e., nonsocial context. The current electroencephalography (EEG) study was designed to explore the role of individual differences in OCS in *social* performance monitoring with a focus on the role of responsibility for other’s harm.

OCD was the first disorder to be investigated in the context of (nonsocial) performance monitoring (Gehring, Himle, & Nisenson, 2000). More than 30 years ago, Pitman (1987) already suggested that compulsions in OCD result from persistent high error signals that cannot be eliminated by behavioral actions. It was only after the discovery of an event-related potential (ERP) component related to error detection (Falkenstein, Hohnsbein, Hoormann, & Blanke, 1990; Gehring, Goss, Coles, Meyer, & Donchin, 1993) that his model could be formally tested. This component, the so-called error-related negativity (ERN), is usually elicited in speeded-choice reaction time paradigms, such as the Flanker task (Eriksen & Eriksen, 1974), and is characterized by a negative frontocentral deflection, which occurs immediately after an incorrect response and reaches its peak 50-100 ms later (Gehring et al., 1993). The ERN has been suggested to result from dopamine-driven prediction errors generated in the anterior midcingulate cortex (aMCC) or the posterior medial frontal cortex (pMFC) more broadly and is thought to trigger subsequent behavioral adjustments and learning (for a theoretical overview see Ullsperger, Danielmeier & Jocham, 2014a). The ERN is accompanied by a later positive component known as the error positivity (Pe). The Pe often is divided in an early and a more centroparietal-oriented late or classical component and is thought to be involved in the conscious affective processing of errors (Ullsperger, Fischer, Nigbur, & Endrass, 2014b). Research on nonsocial performance monitoring has repeatedly demonstrated increased ERN amplitudes in both OCD patients and nonclinical samples scoring high on OCS (see Riesel, 2019 for a recent review and meta-analysis), whereas alterations of the Pe are generally not observed (see Endrass & Ullsperger, 2014).

It has long been recognized that motivational or affective factors and individual differences can modulate ERN amplitudes (Proudfit, Inzlicht, & Mennin, 2013) and performance monitoring more generally (Koban & Pourtois, 2014). For example, Pailing and Segalowitz (2004) demonstrated that higher monetary incentives led to higher ERNs, but this amplitude difference was smaller or absent for individuals high in conscientiousness and low in neuroticism. Similarly, Riesel, Weinberg, Endrass, Kathmann, and Hajcak (2012) showed enhanced ERNs when errors were punished, with larger effects for those with higher trait anxiety. Importantly, however, most studies have been limited to an individual context. Yet, as social beings, humans are continuously in interaction with others. This means that in order to behave in a flexible and adaptive way, we do not only need to take our own but also other people’s actions and the consequences of our own actions for others into account when monitoring our performance (de Bruijn, de Lange, von Cramon, & Ullsperger, 2009). Recent research therefore has ventured into the domain of social performance monitoring to provide a more integrative account of performance monitoring. For example, functional magnetic resonance imaging research using the so-called Cannonball task has demonstrated that performing while being responsible for the outcomes of a co-actor resulted in activation within the dorsal medial prefrontal cortex (dMPFC) (Radke, de Lange, Ullsperger, & de Bruijn, 2011). This area is part of the “mentalizing” network, a network involved in sharing or inferring other’s states (Van Overwalle, 2011), suggesting that participants were concerned with how their performance affected others. Other labs have shown, for example, increased ERNs for errors made while being evaluated by another person (Hajcak, Moser, Yeung, & Simons, 2005) and increased activation of the pMFC and the insular cortex, a brain area associated with the conscious or affective processing of errors, for mistakes that resulted in harm to a friend compared to non-harmful mistakes (Koban et al., 2013). A recent EEG study from our lab additionally showed enhanced ERNs following mistakes that negatively affected a co-actor, but only after administration of the neuropeptide oxytocin (de Bruijn, Ruissen, & Radke, 2017). Oxytocin has been theorized to play an important role in social motivation and salience attribution to social cues (Ma, Shamay-Tsoory, Han & Zink, 2016), suggesting that this compound may have worked to enhance perceived responsibility in the social context. In addition, we recently demonstrated enhanced ERNs for mistakes that had harmful (hearing a loud aversive sound) versus nonharmful (hearing a soft nonaversive sound) consequences for a co-actor (De Bruijn, Jansen & Overgaauw, 2020). These studies suggest that heightened feelings of responsibility and affective distress associated with social mistakes may result in increased performance monitoring and may induce additional social cognitive processes. Additionally, individual differences in responsibility and concern for other’s harm may moderate these processes.

According to the cognitive theory of OCD, inflated perceived responsibility for harm plays a crucial role in the onset of the disorder as patients misinterpret intrusive thoughts as indicating that they are responsible for preventing harm coming to others or oneself and that actions (e.g., compulsions) are needed to prevent feared events from happening (Salkovskis, 1985). It is believed that fear of harm coming to others is a relatively unique feature of OCD, with other anxiety-related disorders (e.g., health anxiety and agoraphobia) better characterized by a fear of harm coming to oneself (Rachman, 2002). The current study therefore takes a first step in examining the role of these social symptoms by investigating how low versus high OCS in a healthy sample are associated with electrophysiological indices of performance monitoring during a Flanker task in different responsibility contexts, i.e., situations where mistakes have negative consequences for 1) oneself (responsibility for self), 2) someone else (responsibility for other), or 3) no one (no responsibility). We expected that individuals with high OCS would display overall increased ERN amplitudes compared with individuals scoring low on these symptoms. In addition, we expected that, relative to individuals with low OCS, those with high OCS would experience mistakes that negatively affect others as more aversive compared with mistakes that affect own outcome and would therefore display particularly increased ERNs for social mistakes, i.e., when responsible for someone else’s outcome.

## Method

### Participants

Participants were preselected based on self-reported OCS in an online survey study of more than 1,200 participants advertised on the Leiden University Research Participation System (SONA). Individuals scoring low (≤9) or high (≥21) on the revised version of the Obsessive–Compulsive Inventory (OCI-R) were invited to take part in the study, based on the suggestion that using a cutoff score of 21 provides the optimal balance between sensitivity and specificity in separating OCD patients from controls (Foa et al. 2002). A total of 56 healthy volunteers between ages 18 and 35 years participated in the experiment. One participant was excluded due to an insufficient number of errors (<6) made in the task, in accordance with the indications by Olvet and Hajcak (2009) that a minimum of 6 trials are needed to obtain reliable ERNs. Two participants were excluded for having made too many errors (>45%) and two other participants were excluded due to poor data quality, leaving a total of 51 participants for analysis. Table 1 displays the characteristics for each group. Users of antidepressants or comparable medication and individuals with a psychiatric diagnosis were excluded from the study. Participants completed the experiment for course credits or monetary compensation and provided written informed consent. The study was approved by the ethics committee of the Institute of Psychology (Leiden University) and was conducted in accordance with the latest version of the declaration of Helsinki.

**Table 1.** Group characteristics of individuals scoring low and high on OCS (means and SDs)

		Low OCS (N = 27)	High OCS (N = 24)	*p* value
Age		20.44 (2.28)	20.42 (2.90)	0.970
Gender (M/F)		4/23	1/23	0.202
Handedness (L/R)		0/27	1/26	0.284
OCI-R	*Washing*	.15 (.46)	2.9 (2.43)	<0.001
	*Checking*	.74 (1.06)	4.58 (2.65)	<0.001
	*Ordering*	.22 (.42)	5.96 (2.68)	<0.001
	*Obsessing*	.89 (1.40)	6.13 (3.17)	<0.001
	*Hoarding*	1.85 (1.35)	6.42 (2.08)	<0.001
	*Neutralizing*	.11 (.32)	2.71 (1.88)	<0.001
	*Total*	3.96 (2.39)	28.71 (1.88)	<0.001
BDI-II		5.37 (4.67)	12.50 (7.06)	<0.001
STAI-T		32.74 (8.42)	45.92 (9.12)	<0.001
RAS	Total	114.69 (20.68)	94.93 (19.46)	0.001
	Self	31.04 (8.05)	26.13 (7.90)	0.033
	Other	34.63 (8.83)	29.33 (9.14)	0.041

OCS = Obsessive-compulsive symptoms; OCI-R = Obsessive-Compulsive Inventory – Revised; BDI-II = Beck Depression Inventory II; STAI-T = State Trait Anxiety Inventory – Trait; RAS = Responsibility Attitude Scale

### Experimental procedure and task

**Fig. 1:**
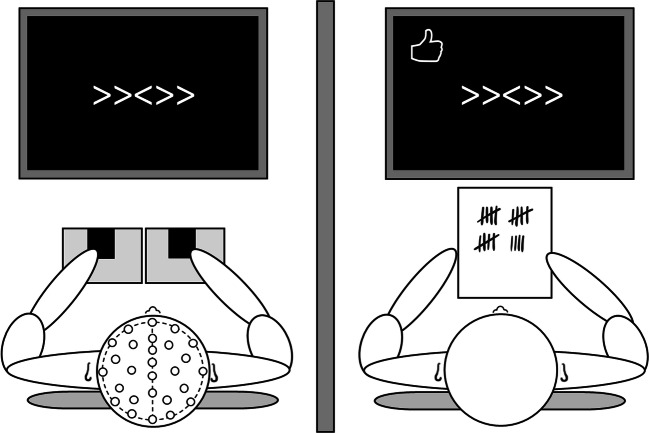
Experimental setup of the Error Responsibility Task. The left participant performed a Flanker task in three different responsibility conditions while EEG was recorded. Meanwhile, the participant on the right counted their mistakes and the number of time warnings

Two participants were invited to the lab. One of these participants was preselected based on OCS and underwent EEG recordings. This participant performed the Error Responsibility task (ERT), a modified version of the Flanker task (Eriksen & Eriksen, 1974) (Figure 1). The goal of this task is to respond with the left or right index finger according to the direction of the middle arrow in a string of five arrows. Half of the trials presented congruent stimuli, i.e., the middle arrow points in the same direction as the surrounding arrows (i.e., <<<<< or >>>>>), and the other half presented incongruent stimuli (i.e., <<><< or >><>>). To ensure that participants made enough mistakes, the task was programmed so that the fixation cross between each trial turned red when less than two errors were detected in the preceding 12 trials. Participants were told that this red cross served as a time warning, indicating that they were responding too slowly and emphasizing the need to speed up. The experimental trials were presented in E-prime (Psychology Software Tools, Inc., Pittsburgh, PA). Each trial started with a fixation cross (450 ms, but 1,000 ms when a red cross was presented), followed by a blank screen (250 ms). The target arrows were presented for 100 ms. Then, a blank screen was presented again for 900 ms during which the participants had time to respond. After this, a blank screen was shown again for 50 ms.

Participants started with two individual practice blocks. Following this practice condition the other participant, i.e., the confederate, came in. The confederate was introduced to the participant and seated behind the computer next to him or her. The confederate was instructed to observe the participant’s performance by counting the number of mistakes made by the participant as well as the number of presented time warnings. To this end, the confederate’s computer screen was mirrored to that of the participant. The confederate could count the mistakes based on a thumbs up or thumbs down sign presented in the top left corner of the screen for each trial, which was not visible on the EEG participant’s screen. Subsequently, the task was performed in three different responsibility conditions, the order of which was counterbalanced across participants. Mistakes of the “responsible player” either 1) did not affect any monetary bonus, 2) affected only their own bonus, or 3) affected only the confederate’s bonus. Crucially, only the preselected participant was responsible for the bonuses and performed the task. In the two responsibility conditions, 20 eurocents was subtracted from either the participant’s or confederate’s bonus from an initial bonus of 10 euros for every mistake and every time warning.

Each condition consisted of two blocks of 120 trials, resulting in a total amount of 960 trials. The task lasted approximately 45 minutes, including short breaks. After each condition, participants were asked to indicate on visual analog scales from 0 to 100 to what extent they 1) felt angry, 2) felt frustrated, 3) disliked making mistakes, 4) felt responsible for their mistakes, and 5) were afraid to make mistakes.

### Measures

All participants completed the OCI-R (Foa et al., 2002). This is an 18-item self-report measure assessing symptoms of OCD compromised of 6 subscales: washing, checking, ordering, obsessing, hoarding, and neutralizing. Each question is answered on a scale from 0 (not at all) to 4 (extremely). The questionnaire has excellent psychometric properties, demonstrated both in patients (Foa et al., 2002) and in a nonclinical college sample (Hajcak, Huppert, Simons & Foa, 2004). To measure general beliefs about responsibility for harm, participants completed the Responsibility Attitude Scale (RAS; Salkovskis et al., 2000). This 26-item questionnaire is rated on a 7-point scale ranging from totally agree (1) to totally disagree (7), with higher scores indicating lower perceived responsibility. The RAS has been reported to have high reliability and internal consistency (Salkovskis et al.). From the original RAS, we additionally took the eight most suited items and rephrased these to create a “self” and “other” version to dissociate between responsibility for harm coming to oneself versus others. For example, in the “self” version, the original item “I often feel responsible for things which go wrong” was rephrased as “I often feel responsible for things which go wrong for myself.” In the “other” version, this was rephrased as “I often feel responsible for things which go wrong for others.” This resulted in two separate, eight-item questionnaires. Participants also completed the State Trait Anxiety Inventory–Trait (Spielberger, Gorsuch, & Lushene, 1970) and the Beck Depression Inventory (BDI-II; Beck, Steer, & Brown, 1996) to assess symptoms of anxiety and depression respectively.

### Electrophysiological recordings and pre-processing

EEG was recorded using an elastic cap with 31 electrodes (midline: Fz, FCz, Cz, Pz, Oz; lateral: AF3-4, FC1-2, FC5-6, F3-4, F7-8, T7-8, C3-4, CP1-2, CP5-6, P3-4, P7-8, PO3-4, O1-2) according to an extended version of the 10-20 system. Vertical and horizontal eye electro-oculograms (EOGs) were recorded from electrodes above and below the right eye and at the outer canthi of the eyes, respectively. Data were acquired with a sampling rate of 512 Hz. Electrodes were referenced to common mode sense (CMS) during data acquisition, and afterwards re-referenced to the average of both mastoids. Data were further processed and analyzed using Brain Vision Analyzer (BVA) version 2 (Brain Products, Munich, Germany). All channels were filtered using a high-pass filter of 0.02 Hz and a time constant of 8 seconds. Subsequently, a lowpass filter of 20 Hz (order 8) and a notch filter of 50 Hz was applied on all channels except the EOG. Before ocular correction, a lenient artifact rejection was performed on all electrode channels using the following settings: maximum allowed voltage step: 50 Hz, maximum allowed amplitude difference: 300 μV in 200-ms interval, minimal and maximum allowed amplitude: −250 and 250 μV, lowest allowed activity in interval: 0.5 μV in 100 ms interval. Subsequently, eye movements were corrected using the automatic independent component analysis (ICA) for ocular correction as provided in BVA and checked afterwards. If for individual cases the automatic ocular ICA correction proved unsatisfactory (e.g., if stimulus-locked cardiac activity was observed), the semiautomatic procedure was performed to remove EOG and cardiac artifacts. After the ocular correction, a stricter artifact rejection was applied by using the following settings: maximum allowed voltage step: 50 Hz, maximum allowed amplitude difference: 100 μV in 200-ms interval, minimal and maximum allowed amplitude: −75 μV and 75 μV. Response-locked ERPs were averaged separately based on condition and correctness and time-locked to response onset, from 200 ms before to 600 ms after the response. These ERPs were subsequently baseline corrected relative to a pre-response duration of 200 ms.

The ERN was determined for correct and incorrect trials separately and quantified as peak-to-peak amplitude at electrode Fz, FCz, and Cz by subtracting the most positive peak in the −80 to 80 ms time window from the most negative peak in the 0 to 150 ms time window (de Bruijn et al., 2017, 2020). For the Pe, we focused on the “late” component, which was defined as the mean amplitude in the 300-500 ms after the response for electrodes Fz, FCz, Cz, and Pz in line with previous research (de Bruijn et al., 2017; de Bruijn et al., 2020).

In line with previous studies (Endrass, Riesel, Kathmann, & Buhlmann, 2014; Riesel, Goldhahn, & Kathmann, 2017a; Riesel et al., 2019b), peak amplitudes were determined with a time interval of 20 ms surrounding each peak in order to reduce the influence of background EEG noise (Clayson, Baldwin, & Larson, 2013).

### Statistical analyses

First, all trials with too fast (<100 ms), too slow (>800 ms), or no responses were removed from the dataset (1.2% of all trials). The presence of standard behavioral Flanker effects was investigated using repeated measures ANOVAs. The first analysis included the within-subject factors congruency (congruent vs. incongruent), condition (no-responsibility, responsibility-for-self, responsibility-for-other) and the between-subject factor OCS (low vs. high) for reaction times to correct responses only. The same factors were used to investigate the error rates. To investigate differences between erroneous and correct trials, reaction times were analyzed using the within-subject factors correctness (correct vs. incorrect) and condition and the between-subject factor OCS. Because erroneous responses to congruent trials are rare, this analysis was performed on incongruent trials only.

For the ERP analyses, ERN and Pe amplitudes were first analyzed for incongruent trials only using correctness, condition and electrode (*ERN*: Fz, FCz, Cz; *Pe*: Fz, FCz, Cz, Pz) as within-subject factors and OCS as between-subject factor to investigate the effect of correctness. Subsequently, we removed the factor correctness to investigate error trials only. In case of sphericity violation, Greenhouse-Geisser corrections were applied. Lastly, visual analog scales with self-reported states were analyzed using repeated measures ANOVAs with condition as within-subject factor and OCS as between-subjects factor.

## Results

### Behavioral data

Table 2 displays the mean reaction times. The analysis on correct responses only showed the expected effect of congruency, F(1,49) = 774.38, *p* < 0.001, η_p_^2^ = 0.94, with slower reaction times for incongruent (314 ms) compared with congruent trials (234 ms). No other significant main or interaction effects were observed (Fs < 1.84, *p*s > 0.165). The analysis on incongruent trials only showed the expected effect of correctness, F(1,49) = 984.44, *p* < 0.001, η_p_^2^ = 0.95. The interaction between condition and correctness did not reach significance, *p* = 0.091. No other significant effects were observed (Fs < 2.07, *p*s > 0.132).

**Table 2.** Mean reaction times in milliseconds for the obsessive-compulsive groups across the different conditions (means and SDs)

	Low OCS (N = 27)			High OCS (N = 24)		
	Congruent	Incongruent		Congruent	Incongruent	
	Correct	Correct	Error	Correct	Correct	Error
No responsibility	236 (29)	321 (39)	221 (27)	233 (32)	312 (50)	222 (41)
Responsible for self	238 (28)	318 (41)	230 (31)	229 (30)	306 (44)	216 (33)
Responsible for other	238 (28)	319 (36)	222 (26)	230 (28)	309 (45)	218 (37)

OCS = Obsessive-compulsive symptoms

Table 3 shows the error rates across the different conditions and OCS groups. The error-rate analysis showed the expected effect of congruency, F(1,49) = 415.18, *p* < 0.001, η_p_^2^ = 0.89, with more errors for incongruent trials (21.4%) compared with congruent ones (1.8%). There also was a main effect of condition, F(1,98) = 4.89, *p* = 0.009, η_p_^2^ = 0.091. Participants made significantly more mistakes in the no-responsibility condition (12.2%) compared with the condition in which they were responsible for the bonus of the other participant (11.0%, *p* = 0.002). The between-subjects effect of obsessive-compulsive group did not reach significance, *p* = 0.10. No interaction effects were observed (Fs < 1.52, *p*s > 0.224). Note that the analyses on post-error slowing did not show any effects of OCS or condition either (see *Supplemental Results*).

**Table 3.** Error rates (%) for the obsessive-compulsive groups across the different conditions (means and SDs)

	Low OCS (N = 27)		High OCS (N = 24)	
	Congruent	Incongruent	Congruent	Incongruent
No responsibility	1.9 (1.8)	20.8 (7.2)	2.4 (2.6)	23.8 (8.3)
Responsible for self	1.2 (1.0)	19.7 (7.9)	2.3 (1.8)	22.8 (8.9)
Responsible for other	1.2 (1.6)	19.1 (6.7)	1.7 (1.6)	22.0 (7.6)

OCS = Obsessive-compulsive symptoms

### Error-related negativity

**Fig. 2:**
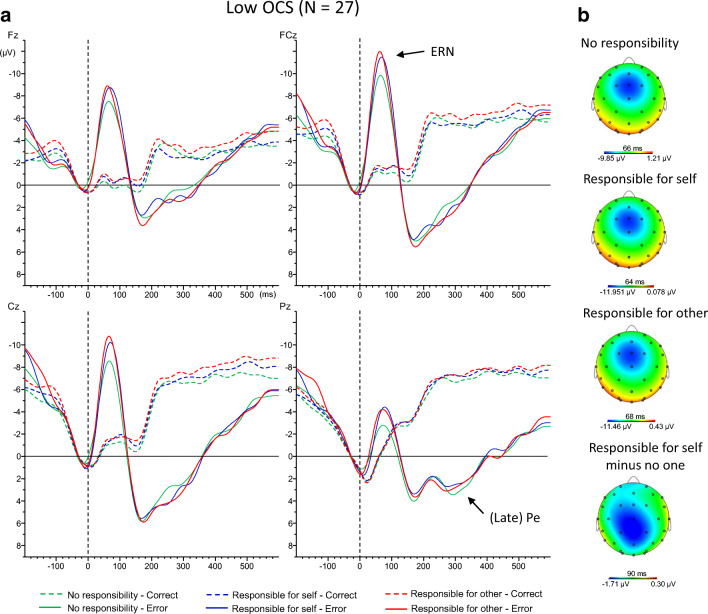
**A**) Response-locked event-related potential waveforms averages for correct and incorrect trials in every condition for the low obsessive-compulsive group at electrode Fz, FCz, Cz, and Pz. A 15-Hz low-pass filter and a −50 to 0 ms baseline correction were applied to the grand averages for visual representation. **B**) Topographical maps of the ERN in the low obsessive-compulsive group at peak onset for each condition as well as for the difference between the responsibility for self and no one condition. OCS = obsessive-compulsive symptoms; ERN = error-related negativity; Pe = error positivity

**Fig. 3.**
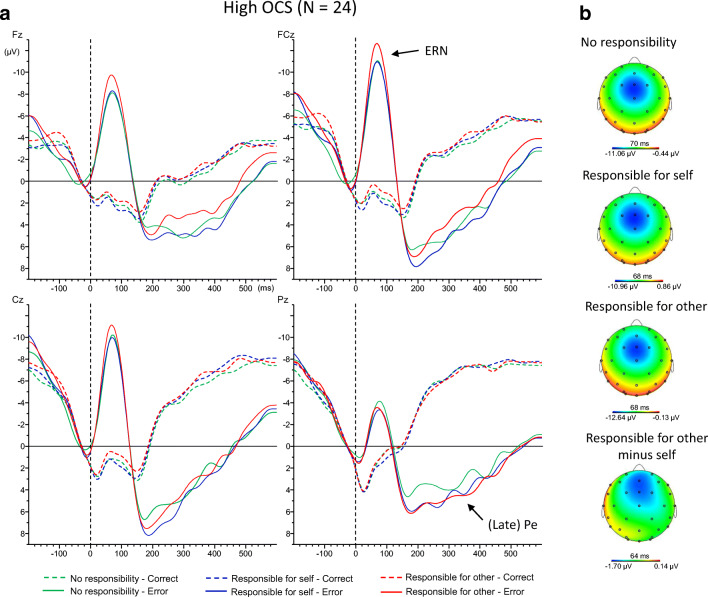
**A**) Response-locked event-related potential waveforms averages for correct and incorrect trials in each condition for the high obsessive-compulsive group at electrode Fz, FCz, Cz, and Pz. A 15-Hz low-pass filter and a −50 to 0 ms baseline correction were applied to the grand averages for visual representation. **B**) Topographical maps of the ERN in the high obsessive-compulsive group at peak onset for each condition as well as for the difference between the responsibility for other and self condition. OCS = obsessive-compulsive symptoms; ERN = error-related negativity; Pe = error positivity

Grand averages of the response-locked waveforms for correct and incorrect trials are displayed in Figure 2 for the low obsessive-compulsive group and Figure 3 for the high obsessive-compulsive group. We first assessed whether the expected main effect of correctness was present using all three (correctness, condition, electrode) within-subject factors. Analysis indeed showed this effect, F(1,49) = 132.71, *p* < 0.001, η_p_^2^ = 0.730, with larger amplitudes for errors (−11.6 μV) compared with correct trials (−3.3 μV). Next, to reduce the complexity of the model and to investigate the error-specificity of possible effects, we removed the factor correctness and focused on error and correct trials separately.

Analyses of error trials only showed a main effect of electrode, F(2,98) = 21.00, *p* < 0.001, η_p_^2^ = 0.300. Amplitudes were largest at FCz (−12.9 μV), followed by Cz (−11.6 μV) and then Fz (−10.4 μV), all *p*s < 0.019, reflecting the frontocentral topography of the ERN (see also Figures 2B and 3B). No main effects of condition or OCS were found (Fs < 1.27, *p*s > 0.285). Neither the interaction between electrode and condition (*p* = 0.141), nor the interaction between condition and OCS (*p* = 0.091) reached significance. There was no significant interaction between OCS and electrode either, *p* = 0.941. However, a significant three-way interaction was observed between electrode, condition, and OCS, F(4,196) = 3.63, *p* = 0.019, η_p_^2^ = 0.069.

Pairwise comparisons focusing on electrode FCz—where error-related negativity (ERN) amplitudes were maximal—showed that participants high in OCS had higher ERN amplitudes in the condition in which they were responsible for the other’s bonus (−14.8 μV) compared with their own bonus (−12.8 μV), *p* = 0.042. This effect was not found for individuals low in OCS (*p* > 0.435). No significant differences with the no-responsibility condition were found (*p*s > 0.145).

Exploratory analyses in the low-scoring group showed a marginally significant effect when comparing the condition in which they were responsible for their own bonus compared with when they were not responsible for any bonus at electrode FCz (*p* = 0.063). This effect reached significance at electrode Cz, −11.7 μV vs. −9.8 μV, *p* = 0.034, indicating that the significant three-way interaction was primarily driven by the two groups displaying different effects of the conditions at these two electrodes.

Analyses of correct trials only showed the expected main effect of electrode, F(2, 98) = 9.30, *p* = 0.001, ηp^2^ = 0.160. Amplitudes were significantly larger at FCz (−3.6 μV) and Cz (−3.5 μV) compared with Fz (−2.8 μV), *p*s < 0.014, but the difference between FCz and Cz was not significant, *p* = 0.372. Importantly, however, no other significant effects were observed (Fs < 1.36, *p*s > 0.261). Table 4 displays the peak amplitudes of the ERN and CRN for the obsessive-compulsive groups across the responsibility conditions at each electrode location.

**Table 4.** Peak amplitudes (μV) of the correct- and error-related negativity for the obsessive-compulsive groups across the responsibility conditions at the different electrode locations (means and SDs)

		Low OCS (N = 27)			High OCS (N = 24)		
		Fz	FCz	Cz	Fz	FCz	Cz
*CRN*	No responsibility	-2.9 (3.3)	-3.8 (3.7)	-3.8 (3.3)	-2.3 (2.5)	-3.1 (3.3)	-3.0 (2.9)
	Responsibility for self	-3.3 (3.6)	-4.2 (3.9)	-3.9 (3.6)	-2.2 (2.1)	-2.9 (2.6)	-2.7 (1.8)
	Responsibility for other	-2.9 (3.6)	-4.1 (3.8)	-4.0 (3.7)	-3.0 (2.6)	-3.7 (3.1)	-3.5 (2.9)
*ERN*	No responsibility	-9.5 (5.5)	-11.3 (7.0)	-9.8 (6.6)	-10.9 (6.3)	-13.5 (7.5)	-12.4 (7.4)
	Responsibility for self	-10.1 (5.8)	-13.0 (6.5)	-11.7 (6.8)	-10.4 (5.7)	-12.8 (6.6)	-11.5 (5.9)
	Responsibility for other	-9.4 (4.4)	-12.3 (5.7)	-11.4 (5.9)	-11.9 (6.3)	-14.8 (7.5)	-13.1 (7.0)

OCS = Obsessive-compulsive symptoms; CRN = correct-related negativity ; ERN = error-related negativity

### Error positivity

Analysis of the late error positivity (Pe) showed the expected effect of correctness, F(1,49) = 73.52, *p* < 0.001, η_p_^2^ = 0.600, with more positive amplitudes for incorrect (4.2 μV) compared with correct trials (−2.2 μV). Next, the factor correctness was removed to reduce the complexity of the model and to investigate the error-specificity of possible effects.

Analysis of error trials separately showed the expected effect of electrode, F(3,147) = 11.77, *p* < 0.001, η_p_^2^ = 0.194. The error positivity was most positive at Pz (5.6 μV), followed by Cz (4.8 μV), FCz (3.6 μV), and Fz (3.0 μV), with only the difference between Pz and Cz not reaching significance (*p* = 0.062). A significant main effect of OCS also was observed, F(1,49) = 8.84, *p* = 0.005, η_p_^2^ = 0.153, showing more positive amplitudes in the high (6.1 μV) compared with the low OCS group (2.3 μV). No other significant effects were present (Fs < 2.00, *p*s > 0.115).

Analyses of correct trials only showed the expected main effect of electrode, F(3, 147) = 24.80, *p* < 0.001, η_p_^2^ = 0.336, showing that amplitudes were most negative at Pz (−7.4 μV) and FCz (−7.1 μV) compared with Cz (−5.2 μV) and Fz (−2.5 μV), *p*s < 0.002, whereas the difference between Pz and FCz was not significant, *p* = 0.561. The between-group effect of OCS was not significant (*p* = 0.339), and no other effects were found, Fs < 1.05, *p*s > 0.372.

### Self-reported states

Means and standard deviations are displayed in Table 5. As expected, a main effect of condition was observed for the question, “I felt responsible for my mistakes,” F(2,98) = 13.85, *p* < 0.001, η_p_^2^ = 0.220, showing that people felt most responsible when they were playing for the other’s bonus compared with when they were playing for their own bonus and when they were not playing for a bonus, *p*s < 0.015. There also was a main effect of OCS, F(1,49) = 8.32, *p* = 0.006, η_p_^2^ = 0.145, showing that participants high in OCS generally felt more responsible for their errors than those low in these symptoms. No interaction between condition and OCS was found (F < 1).

**Table 5.** Self-reported visual analog scores for every condition across the obsessive-compulsive groups (means and SDs)

	Low OCS (N = 27)			High OCS (N = 24)		
	No responsibility	Responsible for self	Responsible for other	No responsibility	Responsible for self	Responsible for other
Anger	26.7 (21.4)	32.4 (23.3)	32.3 (25.5)	21.0 (23.0)	25.9 (26.3)	28.4 (21.8)
Frustration	41.5 (23.1)	48.2 (26.1)	52.3 (24.2)	47.2 (31.9)	50.8 (33.3)	57.33 (28.3)
“I felt responsible for my mistakes”	48.6 (26.1)	57.5 (25.6)	60.6 (23.5)	64.3 (24.7)	72.1 (22.5)	81.2 (19.7)
“I was afraid to make mistakes”	35.0 (23.2)	42.9 (26.9)	46.5 (26.4)	49.9 (27.4)	51.1 (30.0)	69.4 (30.0)
“I disliked making mistakes”	37.2 (27.6)	49.0 (27.2)	55.5 (22.0)	57.5 (28.1)	63.7 (23.0)	77.4 (21.7)

OCS = Obsessive-compulsive symptoms

Participants high in OCS also felt significantly more afraid to make mistakes compared with those low in these symptoms, F(1,49) = 4.85, *p* = 0.032, η_p_^2^ = 0.090. A main effect of condition also was found for this item, F(2,48) = 17.16, *p* < 0.001, η_p_^2^ = 0.417, showing that participants were more afraid when they made mistakes in the condition in which they were responsible for the other’s bonus compared with when they played for their own bonus and compared to the no-responsibility condition, *p*s < 0.001. The difference between the responsibility-for-self and no-responsibility condition did not reach significance (*p* = 0.119). Importantly, an interaction of condition with OCS also was observed, F(2,48) = 4.71, *p* = 0.014, η_p_^2^ = 0.417. This interaction showed that those scoring high on OCS were significantly more afraid to make mistakes when they were responsible for the other’s bonus compared with when they were responsible for their own bonus and compared with the no-responsibility condition, *p*s < 0.001, whereas the difference between the no-responsibility and responsibility-for-self condition was not significant (*p* = 0.767). For those scoring low on OCS, however, the difference between the responsibility-for-self and responsibility-for-other condition was not significant (*p* = 0.269). Participants did report to feel more afraid when they were responsible for the other’s bonus compared with the no-responsibility condition, *p* = 0.006, and scores also were marginally higher for the responsibility-for-self condition compared with the no-responsibility condition (*p* = 0.052). Individuals high in OCS also reported on average to dislike making mistakes to a greater extent than those low in these symptoms, F(1,49) = 9.37, *p* = 0.004, η_p_^2^ = 0.160. A main effect of condition (F(2,98) = 21.49, *p* < 0.001, η_p_^2^ = 0.305) indicated that participants disliked making mistakes most in the condition in which they were responsible for the other’s bonus, followed by the responsibility-for-self condition and the no-responsibility condition (all comparisons *p*s < 0.003). Here, no interaction of group and condition was observed (F < 1).

For anger and frustration, only main effects of condition were found, F(2,98) = 3.13, *p* = 0.049, η_p_^2^ = 0.060 and F(2,98) = 5.31, *p* = 0.006, η_p_^2^ = 0.098, respectively. Both anger and frustration were significantly higher in the responsibility-for-other compared with the no-responsibility condition, *p*s = 0.020, whereas other comparisons did not reach significance (*p*s > 0.080). No effect of group or an interaction with group was found for anger or frustration (Fs < 1).

### Correlations between ERN and self-report measures

**Fig. 4:**
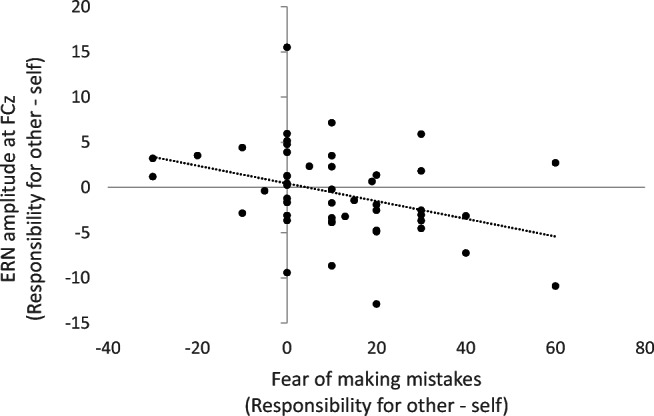
Scatterplot depicting the correlation between the change in ERN amplitudes in μV at FCz between the responsibility for other and self condition and the associated change in fear of making mistakes

To explore to what extent self-reported increases in negative emotions (fear of and dislike of mistakes) in the responsibility-for-other compared with the responsibility-for-self condition were related to changes in ERN amplitudes, we calculated differences scores for these variables. Across all participants, the ∆ERN (responsibility-for-other minus responsibility-for-self at FCz) showed a significant negative correlation with the difference in fear of making mistakes between these conditions (r = −0.362, *p* = 0.009), as well as with the difference in disliking mistakes between these conditions (r = −0.346, *p* = 0.013). However, the latter correlation with disliking mistakes was largely driven by an outlier. When removing this outlier, effects became non-significant (r = −0.237, *p* = 0.098). Figure 4 displays the correlation between the difference in fear of making mistakes and disliking mistakes across these two conditions in relation to the change in ERN. Note that the ERN is a negative ERP component, which means that the negative correlations indicate that relatively larger ERNs in the other versus the self condition are associated with relatively higher fear and dislike of mistakes.

## Discussion

The current study was designed to investigate the role of individual differences in OCS in the electrophysiological correlates of performance monitoring in different social responsibility contexts. Behaviorally, the expected standard Flanker effects were present. Importantly, no differences in performance were detected between the low and high obsessive-compulsive groups. Furthermore, ERP analyses showed that participants high in OCS displayed enhanced ERN amplitudes when they were responsible for the other’s bonus compared with their own bonus. Participants low in OCS instead showed enhanced ERN amplitudes when they were responsible for their own bonus compared with when they were not responsible for any bonus. No between-group differences in ERN amplitudes were found. Finally, participants high in OCS showed larger (i.e., more positive) amplitudes of the late Pe compared with those low in OCS, independent of condition.

On the behavioral level, results are in line with the majority of past performance monitoring research showing an absence of differences in task performance relating to obsessive-compulsive symptomatology or contextual manipulations in the presence of differences on the electrophysiological level (for an overview, see Endrass & Ullsperger, 2014). Behavioral performance did not differ as a function of condition either, with the exception of overall error rates being slightly higher in the no responsibility compared with the responsibility for other condition, something that is likely attributed to the absence of negative (monetary) consequences of making errors in this condition. Because previous research has indicated that differences in performance can affect ERN amplitudes (Fischer, Klein & Ullsperger, 2017), the overall absence of these differences importantly prevents confounding of the electrophysiological results.

ERP results showed that ERN amplitudes importantly differed as a function of OCS across the responsibility contexts. Specifically, participants high in OCS showed enhanced amplitudes of the ERN when they were responsible for the other’s compared with their own bonus. In line with our hypotheses, this finding indicates that high OCS individuals show enhanced monitoring of performance in a social responsibility context, when mistakes negatively affect others instead of the self. Monitoring activity of participants low in OCS, however, did not differ between the condition in which they were responsible for their own bonus compared with someone else’s bonus, indicating that these individuals did not differentiate as much between the two situations. Although not a priori hypothesized, exploratory analyses showed that they did experience a drop in ERN amplitudes in the no-responsibility condition compared with the condition in which they were responsible for their own bonus. This is in accordance with previous research showing that healthy individuals (i.e., individuals scoring low on OCS) are able to downregulate effectively their monitoring activity when less monitoring is needed (Endrass et al., 2010).

Contrary to most previous studies in patients and healthy individuals with low and high OCS (for an overview, see Riesel, 2019), no significant group differences in ERN amplitudes were found. While this was in contrast to our initial hypothesis, we believe that two contextual factors may account for this. In a previous study by Endrass et al. (2010), OCD patients showed enhanced ERNs compared to healthy controls in a standard Flanker task, whereas this difference disappeared when errors were being punished. The authors suggested that the patient group may have been unable to downregulate monitoring activity in situations where less monitoring is required, whereas the healthy controls showed an appropriate upregulation of monitoring activity in the punishment condition. In our study, a monetary punishment was also present, although not in all conditions, which might have led to a similar upregulation. Perhaps more importantly, a confederate was always present thus creating a strong social context. This confederate observed the participant perform the task while counting their mistakes and warnings, inducing an evaluative social element in all conditions. Previous research has indicated that being observed or evaluated by others can lead to enhanced ERNs in healthy volunteers due to errors being perceived as more significant (Hajcak et al., 2005; Voegler et al., 2018). Together, this suggests that the overall motivational and emotional significance of errors in our study may have led to an upregulation of monitoring activity also in the low scoring group, thereby concealing group differences that might have been observed in situations where the need for monitoring is lower.

Overall, participants reported to feel the highest levels of responsibility, fear, and distress about making mistakes when they were responsible for the other’s bonus. Interestingly, increased fear of mistakes in the responsibility-for-other compared with the responsibility-for-self condition also was associated with a relative increase in ERN amplitudes. This is in agreement with a growing body of literature that indicates that the ERN tracks the significance of errors (Proudfit et al., 2013) and that affective and social influences are important determinants of performance monitoring activity (Koban & Pourtois, 2014). Importantly, both the ERN and its neural generator, the aMCC, are known to be modulated by factors, such as anxiety, negative affect, and empathic pain (Koban & Pourtois; Shackman et al., 2011). As the social responsibility context seems to have enhanced levels of fear of mistakes, this presumably increased the need for cognitive or adaptive control, resulting in enhanced ERN amplitudes. The self-reported scale “I felt responsible for my mistakes” however did not correlate with the ERN. This suggests that the feelings of anxiety resulting from being responsible for someone else’s bonus are related to enhanced monitoring rather than the feeling of responsibility itself. Nevertheless, the results indicate that for individuals high in OCS, the social scenario of being responsible for another’s outcome can importantly moderate the magnitude of the ERN and that this is likely attributed to the enhanced emotional significance resulting from this responsibility.

Importantly, participants high in OCS compared with low in OCS felt more responsible for their mistakes, more afraid to make mistakes, and also disliked making mistakes more, independent of responsibility condition. Such a differential appraisal of errors between those low and high in OCS has to our knowledge never been directly demonstrated before and is in line with the notion that obsessive-compulsive symptomatology is associated with increased perceived responsibility (Salkovskis et al., 2000). Note that the current findings also are in line with a previous study by Stern et al. (2010), which showed that OCD patients were significantly more frustrated with their performance and more flustered when making mistakes. Participants with high OCS also reported higher trait levels of perceived responsibility for harm as measured by the RAS, both when it concerned harm coming to others and harm coming to the self. Given the aforementioned evidence that ERNs can be modulated by motivational and affective factors, these results may indicate that previous findings of increased ERNs in OCD patients can in part be attributed to a heightened baseline appraisal of the motivational salience of errors compared to healthy controls. In line with this, research has shown that ERN amplitudes in OCD patients are similar to those of healthy individuals under conditions where the motivational salience of errors is increased, e.g., when errors are being punished (Endrass et al., 2010) or when accuracy is emphasized over speed (Riesel, Kathmann, & Klawohn, 2019a).

In line with the ERN results, participants high in OCS reported higher fear of making mistakes when playing for the other’s compared with their own bonus, whereas fear of mistakes did not differ significantly between these two conditions for individuals low in these symptoms. These results support the idea that individuals high in OCS are characterized by increased levels of responsibility and a fear of making mistakes that affect others (Hezel & McNally, 2016; Salkovskis et al., 2000). Findings thus highlight the fact that the fear of making mistakes that characterizes this group is not limited to harm coming to the self but also is present and even more pronounced when it concerns potential harm to others, which is accompanied by increased monitoring activity as indexed by the ERN. The current findings are particularly important because nearly all previous investigations of performance monitoring and obsessive-compulsive symptomatology focused solely on the individual context, while ignoring the fact that certain social circumstances may moderate and possibly aggravate overactive monitoring.

Unlike the ERN results, we did observe overall group differences in the amplitude of the (late) Pe. Although the exact functional significance of the Pe is still under debate, there is evidence to suggest that this component is associated with the conscious awareness or motivational significance of errors (for a discussion, see Ullsperger et al., 2014a). From this perspective, the enhanced amplitudes observed in the high obsessive-compulsive group seem consistent with the notion that individuals high in OCS are generally more concerned with their errors. In line with the generally higher levels of fear of mistakes in participants with high OCS, previous studies have found a positive relation between the Pe and concern over mistakes (Schrijvers et al., 2009) and have reported reduced Pe in disorder associated with blunted emotional responses, such as psychopathy (Brazil et al., 2009; Maurer et al., 2016) and depression (Schrijvers et al., 2008). It is possible that the social context of this study, where a confederate observing the participant’s task performance was always present, contributed to heightened emotional or motivational salience and thus also increased error awareness of committed errors in individuals with high OCS. Being observed by others can lead to increased self-consciousness and feelings of shame or embarrassment, especially for individuals who are already characterized by high levels of responsibility, perfectionism, anxiety, and worry and who are more focused on preventing harm (Hezel & McNally, 2016). Most previous studies did not observe any differences in Pe amplitudes in relation to obsessive-compulsive symptomatology (Endrass et al., 2008, 2010; Xiao et al., 2011), and the vast majority of OCS-related studies simply do not analyze this component. Importantly, however, these studies have been limited to nonsocial contexts, which highlights the need for replication of this finding.

Note that our stimulus-locked analyses (reported in detail in *Supplementary Materials*) did not reveal any significant (interaction) effects of condition and group for the N1 and P300, supporting the response-locked error specificity of the current findings. However, the analyses did show that the stimulus-locked N2 component was modulated by condition as a function of obsessive-compulsive group. This component is elicited in response to stimulus conflict and has been suggested to be functionally equivalent to the response-locked ERN, because the two components have a similar frontocentral distribution and both indicate a need for adaptive control (Ullsperger et al., 2014a). In line with this, the N2 was recently found to be enhanced in patients with OCD (Riesel, Klawohn, Kathmann & Endrass, 2017b), although previous studies show mixed results (see Riesel et al. for a discussion). In our study, participants with high OCS showed significantly higher N2 amplitudes when they were responsible for the other’s bonus, both compared to the responsibility-for-self condition and compared with those low in OCS. This suggests that, in accordance with the ERN findings, those high in OCS showed particularly enhanced conflict monitoring when they were responsible for the other’s bonus.

In summary, the current outcomes demonstrate that the social context can importantly modulate performance monitoring processes depending on an individual’s level of OCS. Making errors in a social versus an individual context, where errors affected others instead of the self, resulted in enhanced early performance monitoring as indexed by the ERN only in individuals high in OCS. In line with the ERN results, only participants high in OCS reported significantly higher fear of making mistakes when playing for the other’s compared to their own bonus, which underscores the notion that obsessive-compulsive symptomatology is associated with increased levels of concern for how actions might affect others. Enhanced performance monitoring activity in the social compared to individual context was also associated with increased fear of making mistake, supporting existing literature that the subjective salience or distress associated with making errors scales with the magnitude of the ERN. Participants high in OCS also showed higher overall Pe amplitudes, possibly as a result of increased salience and awareness of committed errors under social observation, as well as increased conflict processing as indexed by the N2 specifically when they were responsible for the other’s bonus.

The study has some limitations. First, the sample was predominantly female, while there are indications that gender differences in performance monitoring exist (Fischer, Danielmeier, Villringer, Klein & Ullsperger, 2016). Second, it is unclear to what extent individuals with subclinical obsessive-compulsive symptoms provide a valid analogue for patients with OCD, because these groups may differ on important characteristics. Our findings therefore require replication in more gender-balanced and psychiatric populations. Lastly, it should be recognized that our (response-locked) results could be confounded by individual differences in the amplitude of the stimulus-locked P300 (Meyer, Lerner, De Los Reyes, Laird, & Hajcak, 2017). The employed response-proximal baseline period (−200 to 0 ms) encompasses a large positive shift that is due to the generation of the P300 to the stimulus, which occurs approximately 100 ms before a response. Although no significant differences with regard to the P300 were observed (see *Supplementary Materials*), visual inspection of Figure S4 and S5 of the supplements may suggest some slight variability in amplitudes between our experimental conditions and groups, with a slightly larger P300 for individuals high in OCS. This may for example be due to increased attention in this group (e.g., Polich, 2007). Increased amplitudes of the P300 have previously been observed in patients with OCD, which has been linked to overfocused attention, although reduced amplitudes have been reported as well (for a review see Perera, Bailey, Herring, & Fitzgerald, 2019). Although findings on P300 alterations in OCD are somewhat mixed, it is possible that the P300—e.g., through altered attention allocation—contributed to both the ERN and Pe results. Future research is thus needed to determine to what extent experimental P300 alterations may be responsible for ERN/Pe effects.

## Conclusions

Our findings indicate that high OCS are associated with enhanced performance monitoring in a social responsibility context, when mistakes harm others instead of the self. These results provide an important stepping stone for future studies in patient populations and stress the potential value of taking the social context into account as a way to better understand social symptoms and altered performance monitoring processes in OCD patients. Currently, the ERN is considered an endophenotype of OCD (e.g., Riesel, 2019). Importantly, by gaining more information about the (social) situations in which alterations in the ERN are observed, we might increase classification and risk predictions of and for the disorder and might even discover ways to attenuate or reduce overactive monitoring (Riesel, 2019). Because ERN enhancements are not only observed in OCD, but also in other anxiety-related disorders (see e.g., Meyer, 2016), the social context might help to differentiate these disorders further. For example, while the heightened fear of harming others that characterizes OCD patients predicts enhanced monitoring of performance in a social responsibility context, this might not be expected for other anxiety disorders, such as health anxiety, where fear of harm (i.e., catching a serious illness) is specifically directed towards the self. To conclude, investigating social influences on performance monitoring in different, but related clinical populations may shed important new light on the symptomatology of not only OCD, but also other disorders characterized by altered responsibility attitudes.

## Electronic supplementary material

ESM 1 (DOCX 829 kb)
