# Supplementary material for: Mistakes that matter: An event-related potential study on obsessive-compulsive symptoms and social performance monitoring in different responsibility contexts
Source: Cogn Affect Behav Neurosci. 2020 May 6;20(4):684–97. doi: 10.3758/s13415-020-00796-3 (PMC7394925; doi:10.3758/s13415-020-00796-3)
Supplement: Supplementary file 1 — (DOCX 829 kb) [file 13415_2020_796_MOESM1_ESM.docx]

Supplementary material

**Method**

**Post-error slowing** Post-error slowing is the phenomenon of slowing down after the commission of an error (PES; Rabbitt, 1966). We quantified the PES in accordance with the method proposed by Dutilh et al. (2012) as the difference in reaction time between post-error trials and the corresponding pre-error trials. This quantification is thought to be more robust against global fluctuations in performance compared to more traditional quantifications. Only errors that were preceded and followed by at least one correct response were included in the calculation. PES was investigated using PES (pre-error vs post-error reaction time) and condition as within-subject factors and obsessive-compulsive group as between-subjects factor.

**Stimulus-locked analyses** We calculated stimulus-locked N1, N2 and P300 components for each condition. Segments were created from 200 ms before to 800 ms after the stimulus separately for incongruent and congruent correct trials. The following quantifications were used, in line with previous studies (de Bruijn et al., 2017; de Bruijn et al., 2020). The N1 was defined as the most negative peak occurring in the 150 ms time window after stimulus presentation on congruent trials at FCz, where amplitudes were maximal. The N2 was quantified as peak-to-peak measure at Fz (where amplitudes were maximal) by subtracting the most positive peak in the 150-250 ms time window post response from the most negative peak occurring in the 250-350 ms following stimulus onset (P2/P200) for congruent and incongruent trials separately. The P300 was defined as the most positive peak in the 300 to 800 ms post-stimulus time window for congruent trials at electrode Pz.

**Results**

**Number of trials entering response-locked averages** The mean number of trials included in the averages were as follows: No-responsibility correct = 91 and error = 26, Responsibility-for-self correct = 93 and error = 25, Responsibility-for-other correct = 93 and error = 23. Significantly more correct than incorrect trials entered the average, *p* < .001, but the main effect of condition (*p* = .406) nor the interaction between correctness and condition reached significance (*p* = .067). The interaction between correctness and OCS did not reach significance either (*p* = .085). No other effects were found (Fs < .721, *p*s > .406).

**Post-error slowing** Data of one participant was missing due to the absence of suitable pre- and post-error trials. As expected, a significant main effect of PES was found, F(1,48) = 59.45, *p* < .001, η_p_^2^ = .55, showing that participants were slower after making an error (274 ms) than before (265 ms). No main effect of condition was found, F(2,96) = 1.95, *p* = .229. The interaction between PES and condition was not significant either, F(2,96) = 1.52, *p* = .224. The between-subjects effect of obsessive-compulsive group was not significant, F(1,48) = .88, *p* = .352, and neither were any of the interactions with obsessive-compulsive group (Fs < 1).

**Response-locked grand averages without -50 to 0 ms baseline correction** We applied a -50 to 0 ms baseline correction to the response-locked waveform averages for visualization purposes in the main manuscript, as this better represents our peak-to-peak ERN analyses and outcomes. However, for transparency and completeness, we added the waveform averages using our usually applied -200 to 0 ms baseline correction, as shown in Figure S1 and S2.


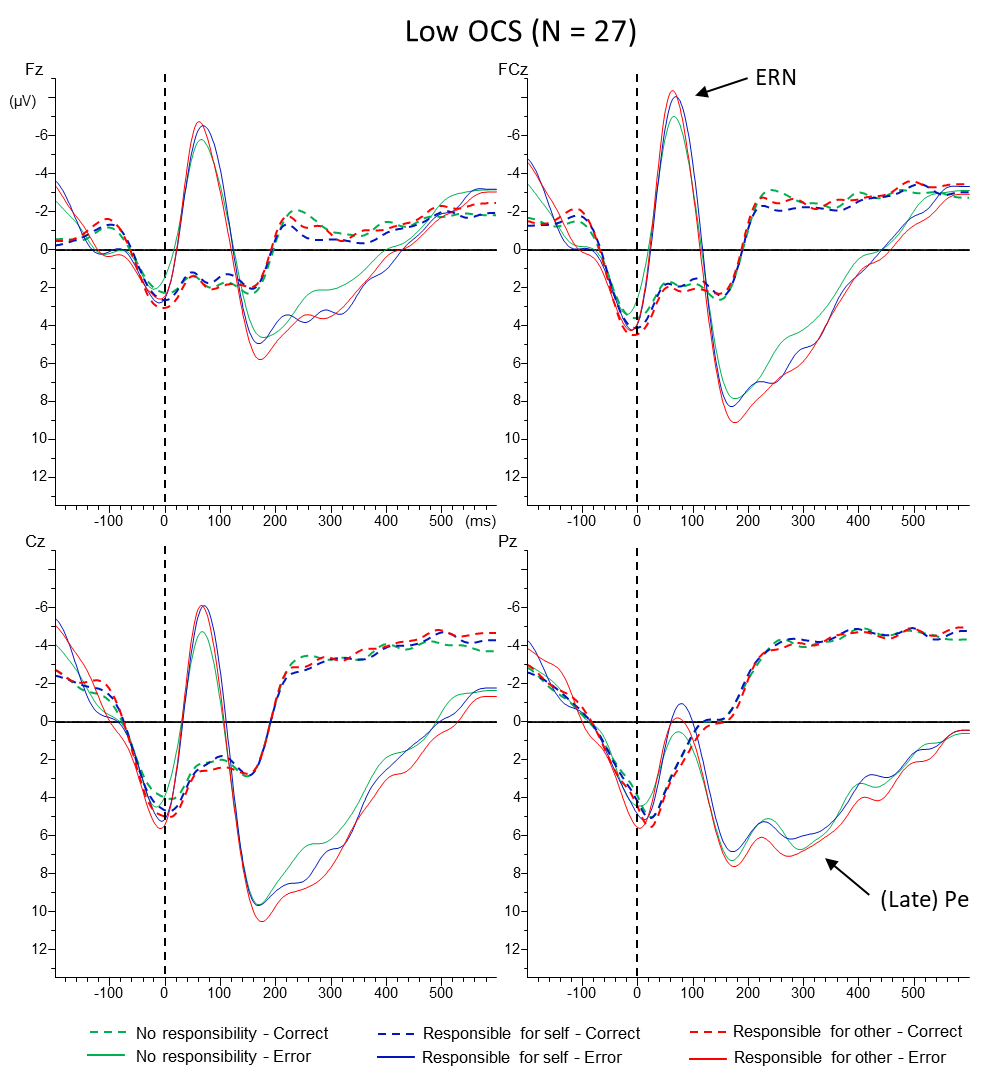


**Figure S1**. Response-locked event-related potential waveforms averages for correct and incorrect trials in every condition for the low obsessive-compulsive group at electrode Fz, FCz, Cz and Pz without a -50 to 0 ms baseline correction. OCS = obsessive-compulsive symptoms; ERN = error-related negativity; Pe = error positivity.

**
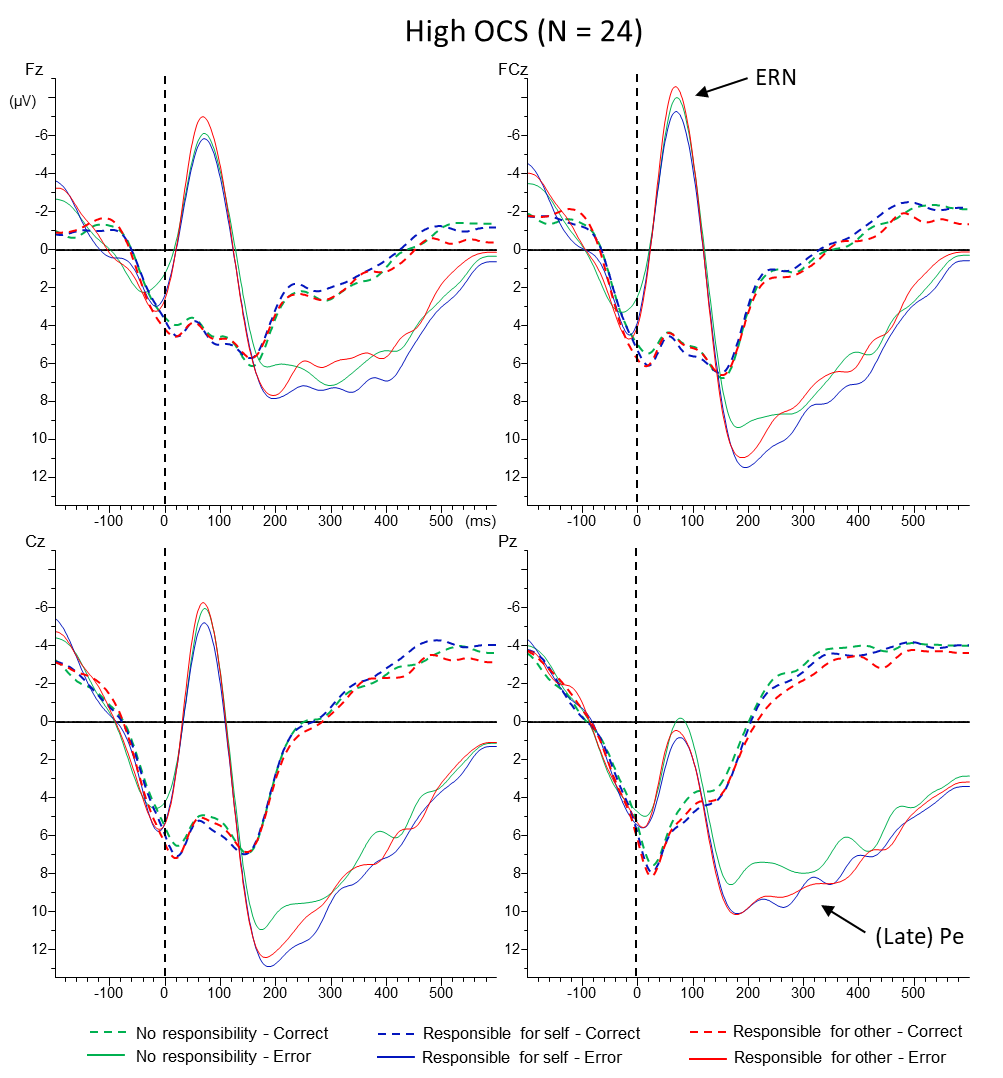
**

**Figure S2**. Response-locked event-related potential waveforms averages for correct and incorrect trials in every condition for the high obsessive-compulsive group at electrode Fz, FCz, Cz and Pz without a -50 to 0 ms baseline correction. OCS = obsessive-compulsive symptoms; ERN = error-related negativity; Pe = error positivity.

**Self-reported states** Figure S3 shows the distribution of self-reported scores for the VAS states ‘I disliked making mistakes’, ‘I felt responsible for my mistakes’ and ‘I was afraid to make mistakes’, separately for the responsibility-for-self versus -other condition and for the low versus high OCS group.

**
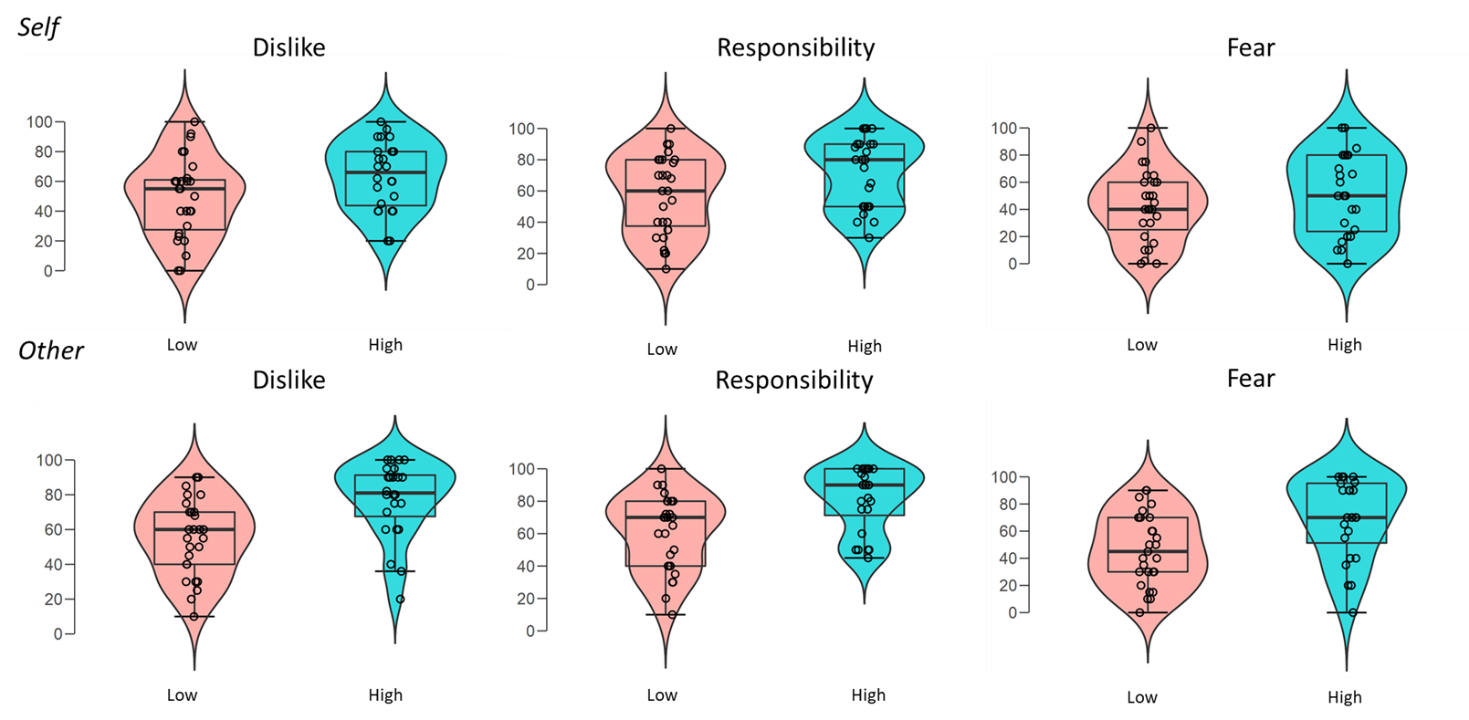
Figure S3.** Feelings about making mistakes for the responsibility for self (top) and other (bottom) condition for the low (red) and high (blue) OCS groups.

**Stimulus-locked results**  Figure S4 and S5 show the stimulus-locked ERPs separately for the low- and high obsessive-compulsive group. The N1 and P300 showed no effect of condition or group nor an interaction with obsessive-compulsive group (Fs < 1.586, *p*s > .214). The N2 showed the expected effect of congruency, F(1,49) = 30.97, η_p_^2^ = .387, with more negative amplitudes for incongruent (-4.2 µV) compared to congruent stimuli (-2.8 µV). Notably, the N2 analysis also showed an interaction of condition and obsessive-compulsive group, F(2,98) = 4.42, *p* = .015, η_p_^2^ = .083. When analyzing congruent and incongruent trials separately, this effect remained significant only for incongruent trials, F(2,98) = 4.54, *p* = .013, η_p_^2^ = .085, but not for congruent trials (*p* = .376). This interaction showed that participants with high OCS showed significantly larger amplitudes on incongruent trials when they were responsible for the other’s bonus (-5.2 µV) compared to when they were responsible for their own bonus (-4.3 µV), *p* = .019. These participants also had marginally higher amplitudes in the no-responsibility (-5.0 µV) compared to the responsibility-for-self condition, *p* = .054. In contrast, participants low in OCS showed marginally larger amplitudes in the responsibility-for-self (-3.8 µV) and the no-responsibility condition (-3.7 µV) compared to the responsibility-for-other condition (-3.2 µV), *p* = .093 and .095, respectively). Participants with high OCS also showed significantly enhanced amplitudes in the responsibility-for-other condition compared to participants low in these symptoms (*p* = .036).


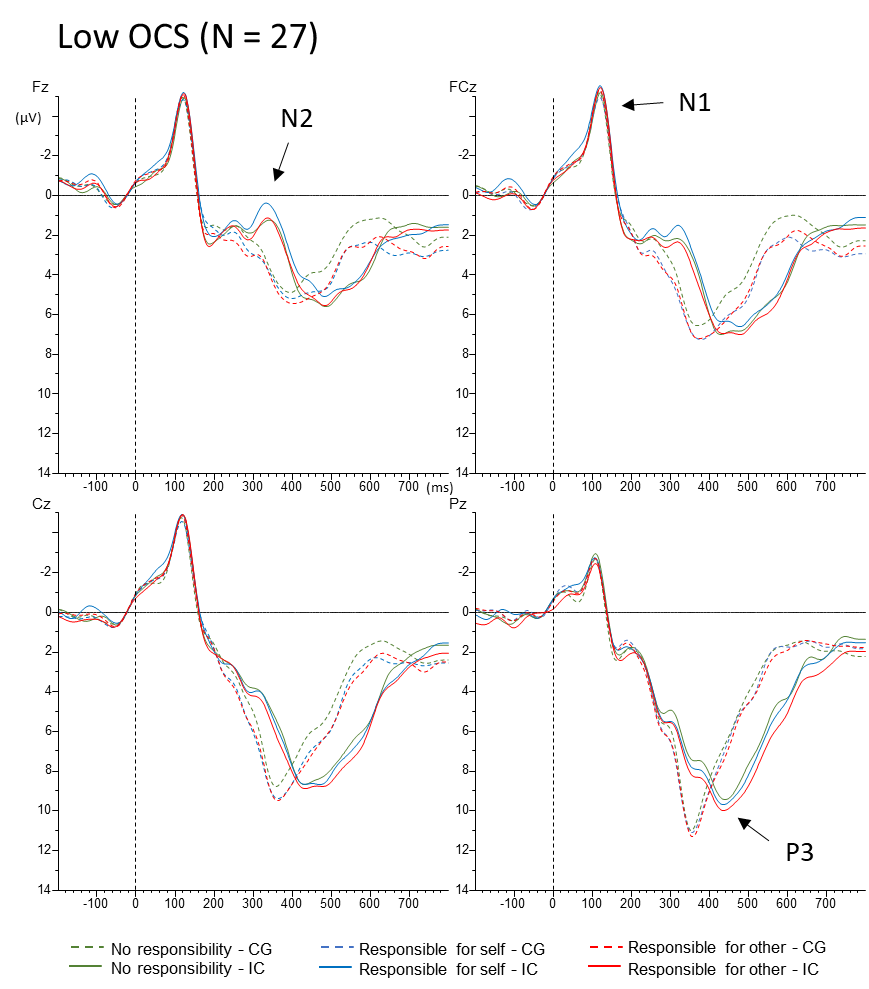


Figure S4. Stimulus-locked event-related potential waveforms averages for congruent and incongruent trials in every condition for the low obsessive-compulsive group at electrode Fz, FCz, Cz and Pz. A 15Hz low-pass filter and a -50 to 0 ms baseline correction were applied to the grand averages for visual representation. OCS = obsessive-compulsive symptoms; CG = congruent; IC = incongruent.


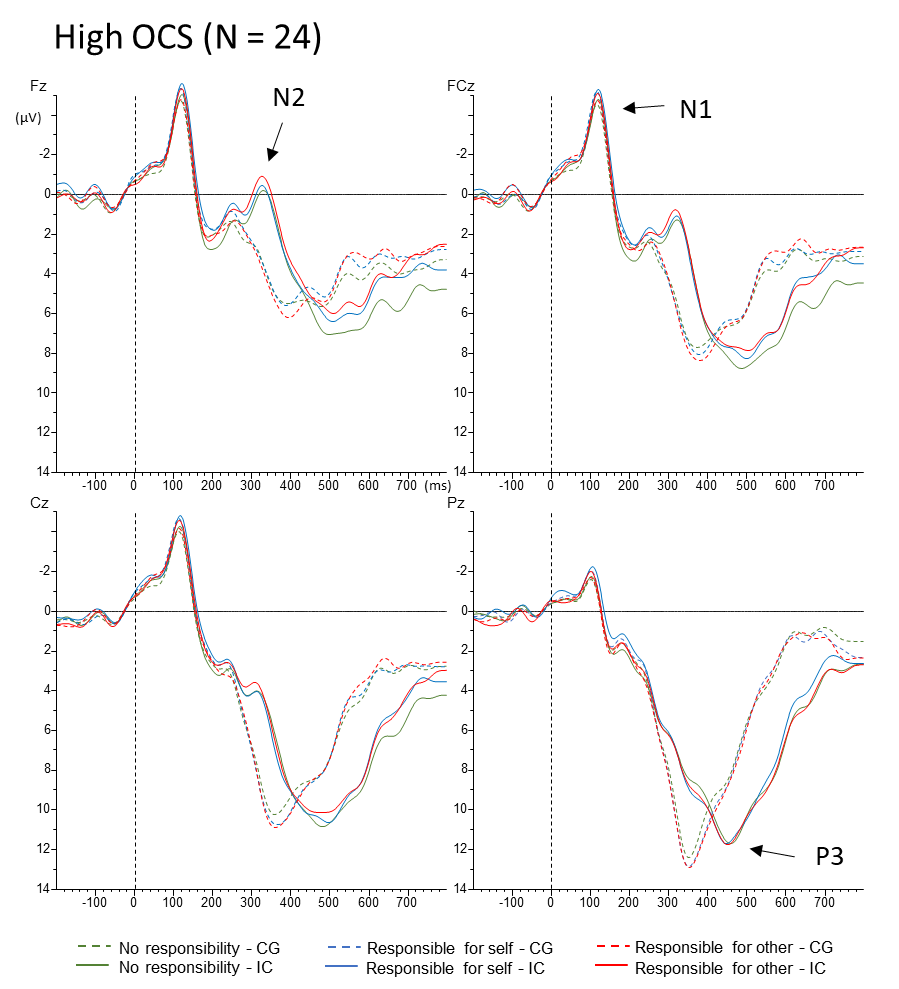


Figure S5. Stimulus-locked event-related potential waveforms averages for congruent and incongruent trials in every condition for the high obsessive-compulsive group at electrode Fz, FCz, Cz and Pz. A 15Hz low-pass filter and a -50 to 0 ms baseline correction were applied to the grand averages for visual representation. OCS = obsessive-compulsive symptoms; CG = congruent; IC = incongruent.

**Correlation between ERN and error rate**

We observed a significant effect of condition on the error rates, showing that participants made significantly more errors in the no-responsibility condition compared to the responsibility-for-other condition (see main manuscript). To investigate the possibility that difference in error rates between these conditions would be accompanied by alterations in ERN/ CRN amplitude, we computed difference scores (responsibility-for-other minus no-responsibility). However, results showed no significant relation between changes in error rates between conditions and ERN nor CRN amplitude at electrode FCz, *r* = .077. *p* = .593 and r = -.056, p = .696, respectively.

**References**

De Bruijn, E. R. A., Ruissen, M. I., & Radke, S. (2017). Electrophysiological correlates of oxytocin induced enhancement of social performance monitoring. *Social Cognitive and Affective Neuroscience, 12*, 1668-1677. <https://doi.org/10.1093/scan/nsx094>

De Bruijn, E. R. A., Jansen, M., & Overgaauw, S. (2020). Enhanced error-related brain activations for mistakes that harm others: ERP evidence from a novel social performance-monitoring paradigm. *NeuroImage, 204*, 116238. <https://doi.org/10.1016/j.neuroimage.2019.116238>

Dutilh, G., van Ravenzwaaij, D., Nieuwenhuis, S., van der Maas, H. L. J., Forstmann, B. U., & Wagenmakers, E. J. (2012). How to measure post-error slowing, a confound and a simple solution. *Journal of Mathematical Psychology, 56*, 208–16. <https://doi.org/10.1016/j.jmp.2012.04.001>

Rabbitt, P.M. (1966). Errors and error correction in choice-response tasks. *Journal of Experimental Psychology, 71*, 264–72. <http://dx.doi.org/10.1037/h0022853>
